# Structural and Functional Insights into Iturin W, a Novel Lipopeptide Produced by the Deep-Sea Bacterium *Bacillus* sp. Strain wsm-1

**DOI:** 10.1128/AEM.01597-20

**Published:** 2020-10-15

**Authors:** Shengnan Zhou, Ge Liu, Rikuan Zheng, Chaomin Sun, Shimei Wu

**Affiliations:** a College of Life Sciences, Qingdao University, Qingdao, China; b CAS Key Laboratory of Experimental Marine Biology, Institute of Oceanology, Chinese Academy of Sciences, Qingdao, China; c Laboratory for Marine Biology and Biotechnology, Qingdao National Laboratory for Marine Science and Technology, Qingdao, China; d Center for Ocean Mega-Science, Chinese Academy of Sciences, Qingdao, China; Nanjing Agricultural University

**Keywords:** *Bacillus*, biocontrol, antifungal, lipopeptide, iturin

## Abstract

In the present study, a deep-sea bacterial strain designated *Bacillus* sp. strain wsm-1 was screened and found to exhibit strong antifungal activity against many plant-pathogenic fungi, and corresponding antifungal agents were thereby purified and determined by tandem mass spectrometry to be two cyclic lipopeptide homologs. These homologs, which were different from any previously reported lipopeptides, were identified to possess identical amino acid sequences of β-amino fatty acid-Asn-Ser-Asn-Pro-Tyr-Asn-Gln and deduced as two novel lipopeptides designated C_14_ iturin W and C_15_ iturin W. Electron microscopy observation indicated that both iturin W homologs caused obvious morphological changes and serious disruption of plasma membrane toward fungal cells, while C_15_ iturin W exhibited more serious cell damages than C_14_ iturin W did, which was well consistent with the results of the antifungal activity assays. To improve the yield and antifungal activity of iturin W, the effects of different carbon and nitrogen sources and amino acids on production of C_14_ iturin W and C_15_ iturin W were investigated. The results indicated that supplements of most of the detected carbon and nitrogen sources could increase the yield of C_14_ iturin W, but inhibit the yield of C_15_ iturin W, while supplements of tryptone and most of the detected amino acids could increase the yield of both C_14_ iturin W and C_15_ iturin W.

**IMPORTANCE** Plant disease caused by pathogenic fungi is one of the most devastating diseases, which affects the food safety of the whole world to a great extent. Biological control of plant diseases by microbial natural products is more desirable than traditional chemical control. In this study, we discovered a novel lipopeptide, iturin W, with promising prospects in biological control of plant diseases. Moreover, the effects of different carbon and nitrogen sources and amino acids on production of C_14_ iturin W and C_15_ iturin W would provide a reasonable basis for the optimization of the fermentation process of lipopeptides. Notably, the structure of iturin W was different from that of any previously reported lipopeptide, suggesting that deep-sea microorganisms might produce many novel natural products and have significant potential in the development of biological products in the future.

## Abstract (precis)

Plant disease caused by pathogenic fungi is one of the most devastating diseases, which affects the food safety of the whole world to a great extent. Biological control of plant diseases by microbial natural products is more desirable than traditional chemical control. In this study, we discovered a novel lipopeptide, iturin W, with promising prospects in biological control of plant diseases. Moreover, the effects of different carbon and nitrogen sources and amino acids on production of C_14_ iturin W and C_15_ iturin W would provide a reasonable basis for the optimization of the fermentation process of lipopeptides. Notably, the structure of iturin W was different from that of any previously reported lipopeptide, suggesting that deep-sea microorganisms might produce many novel natural products and have significant potential in the development of biological products in the future.

## INTRODUCTION

Several microorganisms have been described as potential candidates for biological control agents, and numerous research studies have been focused on members of the genus *Bacillus*. Species from this genus have been considered biologically safe and are commonly used in agriculture (1). Members of the genus *Bacillus* produce a broad spectrum of biologically active molecules, with potential antimicrobial and antifungal properties (2). One of the major factors related to the antifungal activity of members of the genus *Bacillus* is the production of lipopeptides (3–5).

Lipopeptides are low-molecular-weight cyclic amphiphilic oligopeptides with potent antimicrobial activities synthesized by multienzyme complexes called nonribosomal peptide synthetases (NRPSs), which lead to a remarkable heterogeneity among the lipopeptides with regard to the type and sequence of amino acid residues, the nature of the peptide cyclization, and the length and branching of the fatty acid chain (6–8). Lipopeptides produced by the genus *Bacillus* are mainly classified into three families depending on their amino acid sequences: surfactin, iturin, and fengycin. These families share a cyclic β-amino or β-hydroxy fatty acid linked to a cyclic heptapeptide (3, 9). The biological activities of lipopeptides may differ from one compound to another depending on the type of amino acid residues, the cyclization of the peptide, and the length and branching of the fatty acid chain (3, 5).

Iturins are an important class of lipopeptides that have been widely studied for their antifungal activities; they are composed of C_14_ to C_17_ β-amino fatty acids and heptapeptides. The amino acid sequence of the heptapeptide of iturin A is Asn-Tyr-Asn-Gln-Pro-Asn-Ser. Iturins D and E differ from iturin A by the presence of a free carboxyl group in iturin D and a carboxymethyl group in iturin E (10, 11). The iturinic lipopeptides also contain lipopeptides bacillomycin D, bacillomycin F, bacillomycin L, mycosubtilin, and mojavensin, which are mainly different in the amino acid sequences of the heptapeptides (3, 12). In addition, these lipopeptides often contain a number of structural homologs with identical amino acid sequences of heptapeptides and different fatty acid chains (C_14_, C_15_, or C_16_), while the differences of biological activities among these lipopeptide homologs are seldom investigated (13, 14).

The marine environment is a unique habitat, and marine microorganisms exhibit unique metabolic and physiological capabilities conferring on them the ability to produce novel secondary metabolites with biological activities (15, 16). In this study, we purified and identified the novel iturin-like lipopeptide iturin W, which was produced by the marine bacterium *Bacillus* sp. strain wsm-1, isolated from the cold seep in deep sea. This lipopeptide exhibited a broad-spectrum inhibitory effect against many plant fungal pathogens. Furthermore, in order to improve the yield and antifungal activity of *Bacillus* sp. wsm-1 iturin W, the differences of antifungal activities between different homologs were also detected and analyzed in details by electron microscopy, and factors influencing the yield of different homologs were also investigated.

## RESULTS

### Isolation and identification of bacterial strains with strong antifungal activity.

**FIG 1:**
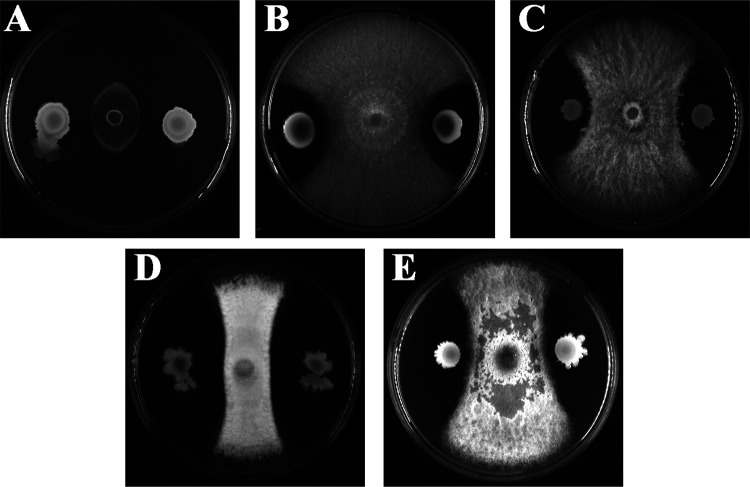
Antifungal activity of Bacillus sp. wsm-1 against different plant pathogens, including Magnaporthe grisea (A), Fusarium solani (B), Fusarium oxysporum f.sp. *lycopersici* (C), *Colletotrichum fioriniae* (D), and Alternaria alternata (E).

In order to get potential strains for biocontrol of plant pathogens, a large number of marine bacterial strains were isolated from the sediment of the deep-sea cold seep, which contain 7 major classes, including *Actinobacteria*, *Alphaproteobacteria*, *Bacilli*, *Betaproteobacteria*, *Clostridia*, *Flavobacteriia*, and *Gammaproteobacteria*. To detect their antifungal activities, plant-pathogenic fungi were preseeded at the center of potato-dextrose agar (PDA) plates, and the isolated bacterial suspensions were inoculated 3 cm away from the preseeded fungi to observe their antagonistic effects at 28°C. As shown in Fig. 1, bacterial strain wsm-1 exhibited the strongest inhibitory effects against most of the detected plant pathogens, including Magnaporthe grisea (Magnaporthales, Magnaporthaceae), Fusarium solani (Sphaeropsidales, Discellaceae), Fusarium oxysporum f.sp. *lycopersici* (Sphaeropsidales, Discellaceae), Colletotrichum fioriniae (Glomerellales, Glomerellaceae), and Alternaria alternata (Pleosporales, Pleosporaceae). Among them, *M. grisea* was the most obviously inhibited. The marine bacterial strain wsm-1 was a rod-shaped, spore-formed, Gram-positive bacterium and formed regular and wrinkled colonies on 2216E agar plates. Its 16S rRNA sequence (MT107176) shared high homology with those of Bacillus velezensis strain CBMB205 (99.78%) and Bacillus amyloliquefaciens strain MPA 1034 (99.78%) in the NCBI database. We further identified the strain by phylogenetic analysis with genes, including 16S rRNA, *rpoD*, and *gyrB*, and the neighbor-joining tree showed that the most closely related neighbors of strain wsm-1 were Bacillus velezensis and Bacillus amyloliquefaciens (see Fig. S1 in the supplemental material). Therefore, the deep-sea bacterial strain wsm-1 was designated *Bacillus* sp. strain wsm-1.

### Purification and identification of antifungal agents produced by *Bacillus* sp. wsm-1.

**FIG 2:**
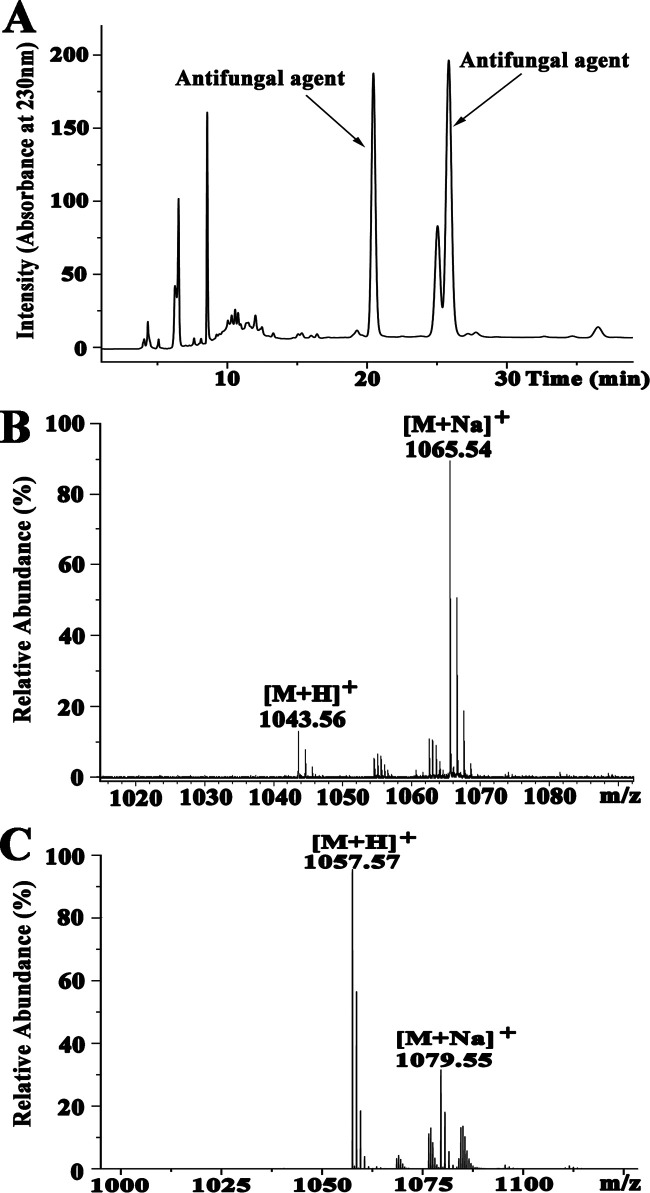
HPLC chromatogram of the antifungal agents produced by *Bacillus* sp. wsm-1 (A) and MS analysis of antifungal agent eluted at 20.451 min (B) and 25.842 min (C).

*M. grisea* was the most obviously inhibited by *Bacillus* sp. wsm-1, so it was selected as an indicator strain during the purification process. The antifungal agents were purified by acidic precipitation, methanol extraction, silica gel column chromatography, and reversed-phase high-performance liquid chromatography (RP-HPLC). At the final purification step, two antifungal agents were obtained at the retention times of 20.451 and 25.842 min (Fig. 2A). In order to determine the molecular mass of the purified antifungal agents, purified fractions were analyzed by mass spectrometry (MS). For the active fraction eluted at 20.451 min, two peaks were detected at *m/z* values of 1,043.56 and 1,065.54 (Fig. 2B), which correspond to the single protonated agent [M+H]^+^ and sodium-cationized ion [M + Na]^+^, respectively. For the active fraction eluted at 25.842 min, two peaks appeared at *m/z* values of 1,057.57 and 1,079.55 with single protonated agent [M+H]^+^ and sodium-cationized ion [M + Na]^+^ (Fig. 2C), respectively. Based on the above-described results, we presumed that the antifungal agent eluted at 25.842 min had one more methylene group (-CH_2_) than the antifungal agent eluted at 20.451 min, which suggested that the two antifungal components were homologs. In combination with our purification scheme and previous reports about iturinic lipopeptides, most of which were detected at *m/z* values of 1,021.49, 1,031.51, 1,043.52, 1,057.53, and 1,071.53, we deduced that the antifungal agents produced by *Bacillus* sp. wsm-1 belonged to the category of iturinic lipopeptides.

### Peptide sequence determination of the antifungal agents produced by *Bacillus* sp. wsm-1.

**FIG 3:**
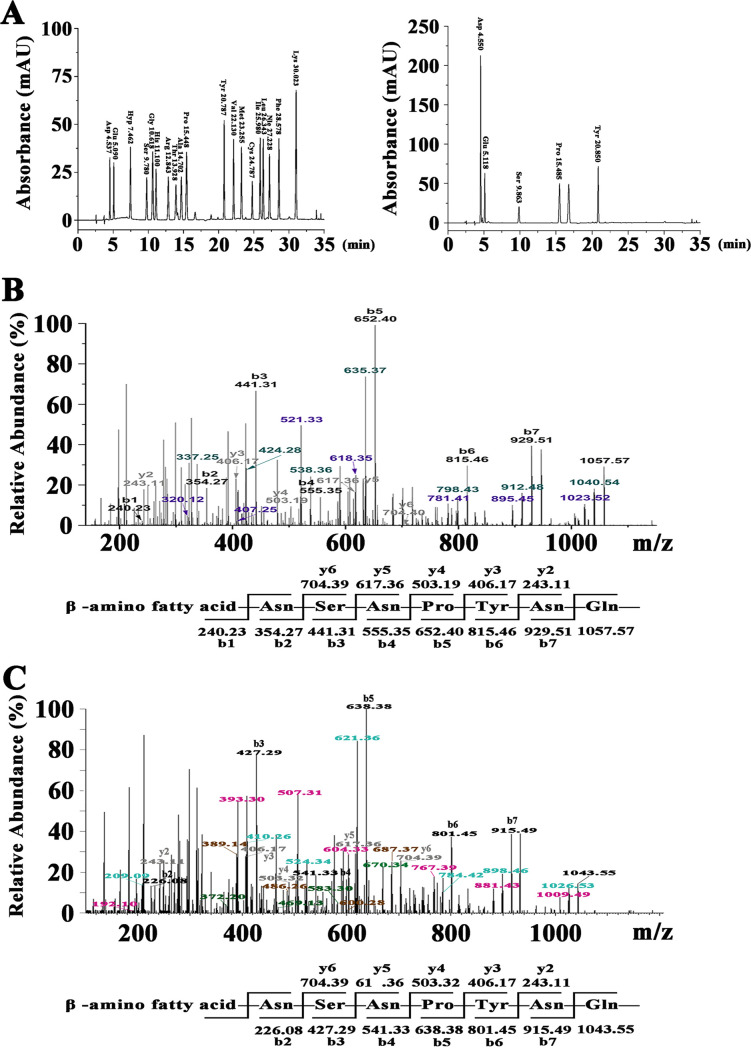
Peptide sequence determination of antifungal agents produced by *Bacillus* sp. wsm-1. (A) Chromatograms of phenylisothiocyanate derivatives of amino acids by RP-HPLC C_18_ with standard amino acids (left) and hydrolyzed sample of antifungal agent eluted at 25.842 min in Fig. 2A (right). (B) MS/MS analysis of the antifungal agent eluted at 25.482 min in Fig. 2A. (C) MS/MS analysis of the antifungal agent eluted at 20.451 min in Fig. 2A.

In order to figure out the amino acid components of the antifungal agents produced by *Bacillus* sp. wsm-1, the main active fraction eluted at 25.842 min was analyzed by amino acid composition analysis. As shown in Fig. 3A, only five amino acids, Asp, Glu, Ser, Pro, and Tyr, were detected, indicating that the antifungal agent contains Asp/Asn, Glu/Gln, Ser, Pro, and Tyr.

To obtain the primary peptide sequences of the antifungal agents produced by *Bacillus* sp. wsm-1, tandem mass spectrometry (MS/MS) was carried out to analyze the antifungal agents eluted at 20.451 min and 25.842 min. As for the antifungal agent eluted at 25.482 min, the b and y fragments by MS/MS analysis are shown in Fig. 3B. Starting from the N terminus, fragments of b ion in order were 929.51 (b7), 815.46 (b6), 652.40 (b5), 555.35 (b4), 441.31 (b3), 354.27 (b2), and 240.23 (b1). Given a value of 1,057.57 for [M+H]^+^, the differences between the values were exactly the mass of ion fragments of Gln, Asn, Tyr, Pro, Asn, Ser, and Asn. Starting from the C terminus, detectable fragments of y ions in order were 704.39 (y6), 617.36 (y5), 503.32 (y4), 406.17 (y3), and 243.11 (y2), and the differences between the values were the mass of ion fragments of Ser, Asn, Pro, and Tyr, which is in accordance with analysis of the fragments of b ion. In combination with the results of the amino acid component analysis as shown in Fig. 3A, the preliminary amino acid sequence of the antifungal fraction eluted at 25.842 min was proposed to be β-amino fatty acid-Asn-Ser-Asn-Pro-Tyr-Asn-Gln from the N terminus to the C terminus.

For the fraction eluted at 20.451 min, as shown in Fig. 3C, starting from the N terminus, fragments of b ion in order were 915.49 (b7), 801.45 (b6), 638.38 (b5), 541.33 (b4), 427.29 (b3), and 226.08 (b2). Given a value of 1,043.55 for [M+H]^+^, the differences between the values were exactly the mass of ion fragments of Gln, Asn, Tyr, Pro, Asn, and Ser. Starting from the C terminus, detectable fragments of y ions in order were 704.39 (y6), 617.36 (y5), 503.32 (y4), 406.17 (y3), and 243.11 (y2), and the differences between the values were the mass of ion fragments of Ser, Asn, Pro, and Tyr, which is consistent with analysis of the fragments of b ion. Thus, the preliminary peptide sequence was proposed to be β-amino fatty acid-Asn-Ser-Asn-Pro-Tyr-Asn-Gln from the N terminus to the C terminus, which is identical to the sequence of antifungal agent eluted at 25.482 min, and indicated that the two antifungal agents were two homologs just with different lengths of fatty acid chains. Therefore, the antifungal fraction eluted at 20.451 min was designated C_14_ iturin W, and the antifungal fraction eluted at 25.842 was designated C_15_ iturin W. Taken together, the amino acid sequences of C_14_ iturin W and C_15_ iturin W were different from previously reported lipopeptides (3, 14), indicating that they are two novel iturinic homologs.

### Antifungal activity assays of C_14_ iturin W and C_15_ iturin W against plant pathogen *M. grisea*.

**FIG 4:**
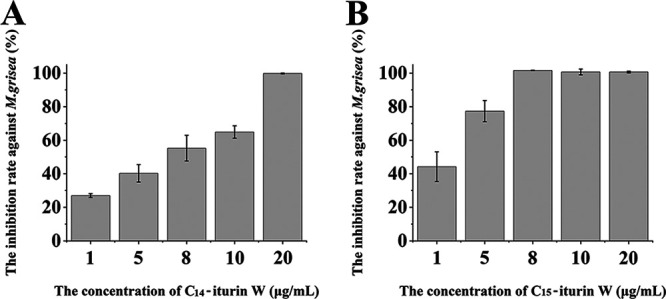
Growth inhibition assay of antifungal agents C_14_ iturin W (A) and C_15_ iturin W (B) against *M. grisea*.

In order to investigate whether there is a difference in antifungal activity between different lipopeptide homologs, the antifungal activities of C_14_ iturin W and C_15_ iturin W were tested against *M. grisea*. As shown in Fig. 4A, when *M. grisea* was treated with 1 μg/ml of C_14_ iturin W, the inhibition rate against *M. grisea* was only 22% and slightly increased as the concentration of C_14_ iturin W increased, and the growth of *M. grisea* was completely inhibited when the concentration of C_14_ iturin W reached 20 μg/ml. However, when *M. grisea* was treated with 1 μg/ml of C_15_ iturin W, the inhibition rate was up to 44% and obviously increased as the concentration of C_15_ iturin W increased, and the growth of *M. grisea* was completely inhibited when the concentration of C_15_ iturin W reached only 8 μg/ml (Fig. 4B), which was much lower than that of C_14_ iturin W against *M. grisea*. Therefore, the antifungal activity of C_15_ iturin W was significantly higher than that of C_14_ iturin W.

### Ultrastructural and morphological changes of *M. grisea* hyphae caused by C_14_ iturin W and C_15_ iturin W.

**FIG 5:**
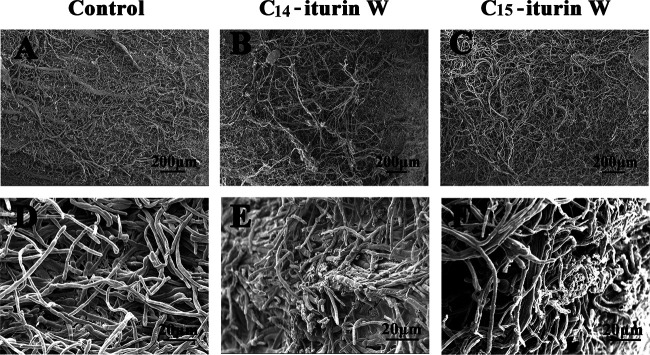
Morphological changes of *M. grisea* hyphae caused by C_14_ iturin W and C_15_ iturin W observed by SEM. The pathogenic fungus *M. grisea* was treated with C_14_ iturin W (B and E) or the same concentration of C_15_ iturin W (C and F), and *M. grisea* was treated with the same amount of methanol in the control group (A and D).

In order to investigate the effects of lipopeptide C_14_ iturin W and C_15_ iturin W on plant pathogen *M. grisea* at the ultrastructural level, *M. grisea* was treated with the same concentration of C_14_ iturin W and C_15_ iturin W and then observed under scanning electron microscopy (SEM) and transmission electron microscopy (TEM). As observed under SEM, the hyphae of *M. grisea* in the control group grew normally from the center to the edge of the colony (Fig. 5A) and exhibited a smooth and straight appearance (Fig. 5D), while the hyphae grew disorderly (Fig. 5B and C) and became rough and broken (Fig. 5E and F) after treatment with C_14_ iturin W or C_15_ iturin W.

**FIG 6:**
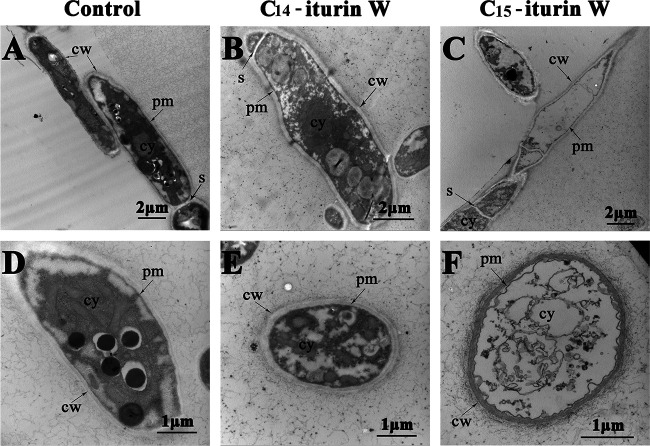
Ultrastructural changes of *M. grisea* hyphae caused by C_14_ iturin W and C_15_ iturin W observed by TEM. The pathogenic fungus *M. grisea* was treated with C_14_ iturin W (B and E) or the same concentration of C_15_ iturin W (C and F), and *M. grisea* was treated with the same amount of methanol in the control group (A and D). cw, cell wall; cy, cytoplasm; pm, plasma membrane; s, septum.

When observed under TEM, the hyphae in the control group showed intact cells with a smooth surface, uniform and darkly stained cytoplasm, and the cellular organelles in normal arrangements (Fig. 6A and D), while the cytoplasm became lightly stained and organelles were missing when *M. grisea* was treated with C_14_ iturin W (Fig. 6B and E), indicating that the plasma membrane was disrupted. The plasma membranes were severely damaged and separated from cell walls, and the cytoplasm almost leaked out completely when *M. grisea* was treated with C_15_ iturin W (Fig. 6C and F). These results indicated that iturin W exerted its antifungal activity primarily by disruption of the plasma membrane, leading to cytoplasm leakage and cell death, which was in accordance with previous reports about antagonistic mechanism of iturinic lipopeptides (17–19). Based on the observation by electron microscopy, we concluded that the antifungal activity of C_15_ iturin W was higher than that of C_14_ iturin W, which was coincident with the results of our above-described antifungal activity assays.

### Medium optimization for the production of lipopeptide iturin W.

**FIG 7:**
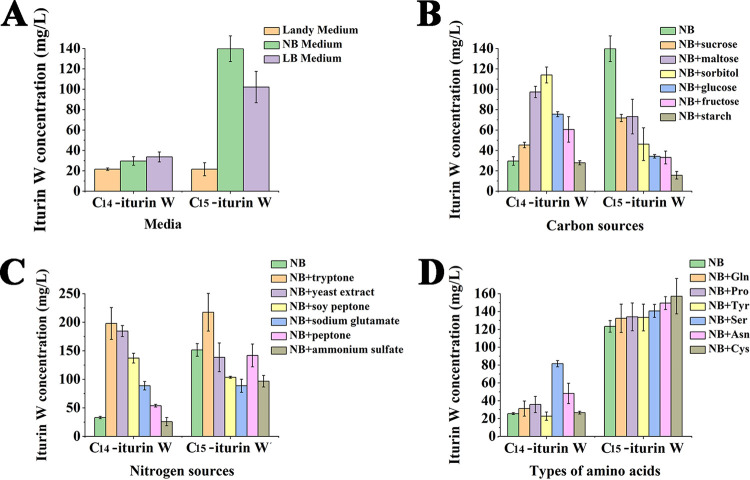
Medium optimization for the production of C_14_ iturin W and C_15_ iturin W. (A) The production of C_14_ iturin W and C_15_ iturin W on different basic media. (B to D) The production of C_14_ iturin W and C_15_ iturin W on NB medium supplemented with different carbon sources (B), nitrogen sources (C), and amino acids (D).

To get more insight regarding the production of lipopeptide iturin W, the cell growth and the production of iturin W by *Bacillus* sp. wsm-1 were determined. The results showed that iturin W was only detected at the stationary phase after *Bacillus* sp. wsm-1 was incubated for 24 h, and the yield reached maximum after incubation for 48 h. In addition, *Bacillus* sp. wsm-1 did not form spores until it was incubated for 24 h, and it formed more spores after it was incubated for 48 h, which indicates that there is a strong correlation between sporulation and lipopeptide production, as previously report by Ambrico et al. (20). In order to obtain a higher yield of iturin W, Luria-Bertani (LB) medium, nutrient broth (NB) medium, and Landy medium were chosen to culture the iturin W-producing strain *Bacillus* sp. wsm-1, and the yields of C_14_ iturin W and C_15_ iturin W were measured after 2 days of incubation. As shown in Fig. 7A, NB medium was more suitable for production of C_14_ iturin W and C_15_ iturin W than LB medium and Landy medium.

The effects of different factors on production of C_14_ iturin W and C_15_ iturin W were further measured based on NB medium. As for the effects of carbon sources on the yield of C_14_ iturin W and C_15_ iturin W, the only significant increases were seen for the yield of C_14_ iturin W, observed with all detected carbon sources except starch. Especially for sorbitol, the yield of C_14_ iturin W was increased more than 3.8-fold. However, the yield of C_15_ iturin W was obviously inhibited when a different carbon source was supplemented (Fig. 7B), which was not really what we expected since the antifungal activity of C_14_ iturin W was much lower than that of C_15_ iturin W. As for the effects of nitrogen sources on production of C_14_ iturin W and C_15_ iturin W, the detected nitrogen sources could increase the production of C_14_ iturin W except ammonium sulfate, while the production of C_15_ iturin W was slightly inhibited except with tryptone. Supplementation with tryptone not only could clearly increase the yield of C_14_ iturin W but also could obviously increase the yield of C_15_ iturin W (Fig. 7C), indicating that tryptone was the most suitable nitrogen source for enhancing the production of iturin W. The effects of amino acids on the yields of C_14_ iturin W and C_15_ iturin W were also detected, and the results showed that most of the detected amino acids could increase the yield of C_14_ iturin W and C_15_ iturin W (Fig. 7D).

## DISCUSSION

Iturins and closely related lipopeptides constitute a family of antifungal compounds known as iturinic lipopeptide, which has strong antifungal activity and has been widely studied for biocontrol of plant pathogens (1, 21). Iturin W, produced by the marine bacterium *Bacillus* sp. wsm-1, exhibited very strong antifungal activity against many plant pathogens, especially *M. grisea*. In our study, C_14_ iturin W and C_15_ iturin W completely inhibited the growth of *M. grisea* at 20 μg/ml and 8 μg/ml, respectively, while the MIC of iturin A against *M. grisea* was reported at 125 μg/ml (22), and iturins 1 and 2/maribasins A and B were reported to possess weak broad-spectrum activity against Alternaria solani, Fusarium oxysporum, Fusarium graminearum, and Verticillium albo-atrum at the MIC values of 25 to 200 μg/ml (2). Therefore, compared with previously reported iturinic lipopeptides, iturin W is a very good prospect for biological control of plant diseases, especially for rice blast disease caused by *M. grisea*.

Iturin is a lipoheptapeptide, and the typical amino acid sequence of the heptapeptide is Asn-Tyr-Asn-Gln-Pro-Asn-Ser (3). Normally, the first three amino acids of heptapeptide of iturinic lipopeptide are conserved, while the remaining four amino acids are variable (10). Notably, based on the fragments of b ions and y ions, the amino acid sequence of heptapeptide of iturin W was deduced as Asn-Ser-Asn-Pro-Tyr-Asn-Gln, which differs from any previously reported iturinic lipopeptides not only in the four variable amino acids but also in the usually conserved second amino acid. In order to verify the amino acid sequence of the heptapeptide of iturin W, we also analyzed the related b ion fragments of C_15_ iturin W in the order 1,040.54, 912.48, 798.43, 635.37, 538.36, 424.28, and 337.25 (shown in green in Fig. 3B) or related b ion fragments of C_14_ iturin W in the order 1,023.52, 895.45, 781.41, 618.35, 521.33, 407.25, and 320.12 (shown in purple in Fig. 3B), which further confirms that iturin W is a novel lipopeptide. In addition, corresponding ion fragments of C_14_ iturin W were also observed. The novel structure of iturin W may due to the unique habitat of marine bacteria, which confers on them the potential to produce novel secondary metabolites with new biological activities.

Most *Bacillus* spp. can produce one type of lipopeptide, and a few can produce two or three types of lipopeptides (23–25). Furthermore, each type of lipopeptide often contains several homologs with identical amino acid residues and different lengths of fatty acid chains (13, 26–28). It has been reported that even though amino acid sequences of surfactin homologs are identical, the biological activities of surfactin homologs are different just because of different alkyl chains (29, 30). In this study, the antifungal activity of C_15_ iturin W was much higher than that of C_14_ iturin W, which further confirmed that the fatty acid chains were critical for determining the biological properties of lipopeptides.

Until now, most of the research has been focused on the structure and function of lipopeptides and their potential applications in biomedical, agricultural, food, and environmental aspects (31–33). Recently, more and more research has been shifting to improve the yield of lipopeptides by optimization of medium components, phenotypic dissociation, genome shuffling, and related metabolic engineering (20, 34, 35). Carbon and nitrogen sources and trace elements have all been demonstrated to exhibit significant influence on the yields of lipopeptides during optimization of the fermentation process (36, 37). Since the activities of lipopeptide homologs are different, it is advisable to enhance the yield of homologs with higher activity rather than enhancing the whole yield of the lipopeptides. In our study, most of the supplemented carbon or nitrogen sources could increase the yield of C_14_ iturin W, but the yield of C_15_ iturin W, with higher antifungal activity, was inhibited to different extents. Fortunately, tryptone supplementation could obviously increase the yields of both C_14_ iturin W and C_15_ iturin W, and the total yield of iturin W was increased from about 180 mg/liter up to 400 mg/liter, which is higher than that of the previously reported iturin A (34, 35). In brief, supplementation of different nutrient sources could not only change the yields of different lipopeptide homologs but also change their final proportions, which will provide a reasonable basis for the optimization of fermentation process of lipopeptides in the future.

## MATERIALS AND METHODS

### Strain isolation, culture conditions, and strain identification.

Marine sediment was collected by RV *KEXUE* from the cold seep in the South China Sea (119°17′04.956”E, 22°06′58.384′′N) at a depth of approximately 1,143 m in September 2017. The marine bacterial strains used in this study were isolated from the samples via the dilution method as described previously (38) and cultured in modified Zobell 2216E broth (5 g/liter of tryptone, 1 g/liter of yeast extract, 1 liter of filtered seawater, pH adjusted to 7.4 to 7.6) at 28°C. The single colonies were further screened for the ability to produce antifungal agents. The plant-pathogenic fungal strains used in this study were incubated onto potato-dextrose agar (PDA; potato at 200 g, glucose at 20 g, agar at 15 to ∼20 g, distilled water at 1,000 ml [natural pH]) and incubated at 28°C. Identification of the isolated marine strain was determined by sequencing 16S rRNA genes. Universal primers 27F (5′-AGAGTTTGATCCTGGCTCAG-3′) and 1492R (5′-TACGGCTACCTTGTTACGACTT-3′) specific for bacterial 16S rRNA genes were used to amplify the corresponding genes. The resultant DNA sequence was compared with known bacterial 16S rRNA sequences in the National Center for Biotechnology Information database (NCBI GenBank) using the Basic Local Alignment Search Tool (BLAST) algorithm.

### Screening of bacterial strains with strong antifungal activity.

To screen marine bacterial strains with high antifungal activity, five important plant fungal pathogens, Magnaporthe grisea, Fusarium solani, Fusarium oxysporum f.sp. *lycopersici*, *Colletotrichum fioriniae*, and Alternaria alternata, which infect and cause significant yield loss in many crops, were selected as indicator strains. Determination of the antifungal activity of isolated strains was carried out as described by Gu et al., with minor modification (39). Briefly, 0.6-cm-diameter plugs containing mycelia of indicator strains were preseeded at the center of the PDA plates and then 5 μl of bacterial suspensions of overnight cultures was inoculated 3 cm away from the fungi on the surface of PDA plates, and the plates were incubated at 28°C for 48 h. The inhibition zones of the fungal growth were determined by observing visually the presence (growth) or absence (nongrowth) of fungi.

### Isolation and purification of antifungal agents from *Bacillus* sp. wsm-1.

To obtain the active agents inhibiting the growth of fungal cells, the procedure was carried out according to the previously described method, with little modification (29). Briefly, an overnight culture of *Bacillus* sp. wsm-1 was inoculated into 100 ml of the fermentation medium in a 250-ml flask and was cultured with shaking at 160 rpm for 48 h at 28°C. The cell-free culture supernatant was obtained by centrifugation at 6,000 × *g* at 4°C for 20 min, subsequently acidified with 6 M HCl to pH 2.5, and stored overnight at 4°C. The resulting precipitate was collected by centrifugation at 4°C and 6,000 × *g* for 20 min, then washed with 30 ml of distilled water, and finally extracted with 10 ml of methanol to get the antifungal agents. The resulting methanol extract was purified through a silica gel column using different ratios of methanol and methylene chloride, and the resulting antifungal fraction was collected and concentrated. The concentrated active fraction was further purified by reversed-phase high-performance liquid chromatography (RP-HPLC; Agilent 1260, USA) with an Eclipse XDB-C_18_ column (5 μm; 4.6 by 250 mm; Agilent, USA). The column was eluted at a flow rate of 2 ml/min with mobile phase A and mobile phase B under the following conditions: 0 to 5 min, 0% mobile phase B to 70% mobile phase B, and 6 to 35 min, 70% mobile phase B, followed by 100% mobile phase B, wherein mobile phase A was composed of water and methanol (90:10, vol/vol) and mobile phase B was 100% methanol. The elution was monitored using a UV detector set at 230 nm.

### Analysis of amino acid component of the purified antifungal agents.

To obtain the amino acid component of the purified antifungal agent, the active fraction of the main peak was harvested from its HPLC fraction, dried by vacuum freezing, and then analyzed according to previously described methods (29). Briefly, the dried sample was hydrolyzed with 1 ml of 6 M HCl at 110°C, then dried, and dissolved in 1 ml of double-distilled water. Eighteen amino acids (Sigma-Aldrich, Saint Louis, MO) were used for standard reference: Asp, Glu, Ser, Gly, His, Arg, Thr, Ala, Pro, Tyr, Val, Met, Cys, Ile, Leu, Phe, Trp, and Lys. A 200-μl aliquot of the 18 standard amino acids (2.5 mM) or the redissolved hydrolysis sample solution was mixed with 100 μl of 0.1 M phenylisothiocyanate and 1 M triethylamine in acetonitrile and then extracted with *n*-hexane. The resulting acetonitrile phase was collected and further analyzed by HPLC on a C_18_ column (5 μm; 250 by 4.6 mm; Agilent, USA).

### Mass spectrometry analysis of the purified antifungal agents.

Mass spectra of active antifungal substances were analyzed by a linear ion trap Orbitrap spectrometer (LTQ Orbitrap XL; Thermo Fisher, USA) using high-energy-collision-induced dissociation (HCD), which is a new mass spectrometry pyrolysis technology and could provide abundant fragmentation information (40). Data from HCD MS/MS were acquired under the following conditions: electrospray ion source (ESI); spray voltage, 3 kV; ion transfer capillary temperature, 275°C; dry gas, nitrogen; pressure, 0.05 mPa; HCD collision gas, helium; anion pattern detection; collision energy of HCD, 30 to ∼40 eV. The results were analyzed by Xcalibur 2.1 (Thermo Fisher).

### Assay of activity of the purified antifungal agents against *M. grisea*.

To determine the activity of purified antifungal agents, the growth inhibition assays against *M. grisea* were performed in 96-well microtiter plates as described by Romano et al., with minor modification (41). Briefly, the bioassay was conducted in a 96-well plate which contained 10 μl of different concentrations of purified antifungal agent and 200 μl of the conidial suspension (3 × 10^4^ spores/ml in potato-dextrose broth [PDB]: potato at 200 g, glucose at 20 g, distilled water at 1,000 ml [natural pH]) of *M. grisea* in each well. Plates were incubated at 28°C on a rotary shaker (160 rpm) for 2 days, and the fungal growth was determined spectrophotometrically at 595 nm by a microplate reader (Infinite M1000 Pro; Tecan, Mannedorf, Switzerland). The growth inhibition rate was calculated as the growth of *M. grisea* treated with the different concentrations of purified antifungal agents normalized to that in the control group treated with equal amounts of methanol. Each treatment was replicated four times, and each experiment was repeated at least two times.

### Ultrastructural and morphological observation of fungal hyphae caused by the purified antifungal agents.

In order to investigate the changes of *M. grisea* caused by antifungal agents, SEM and TEM were used to observe the morphological changes of hyphae of *M. grisea* after treatment with the same concentrations of antifungal agents. Twenty-microliter volumes of purified antifungal agents at a concentration of 1 mg/ml were spotted on sterilized filter paper and placed 2 cm away from the margin of freshly grown *M. grisea*. For scanning electron microscopy, *M. grisea* treated with purified antifungal agents was centrifuged and prefixed with 2.5% glutaraldehyde. Fixed cells were rinsed three times for 10 min with 10 mM phosphate buffer and dehydrated through an ethanol gradient. The samples were then coated with gold and analyzed with a Hitachi S-3400N SEM (Hitachi, Tokyo, Japan). For transmission electron microscopy, samples were embedded in Epon 812 and sectioned using an ultramicrotome, and ultrathin sections were collected and observed at 120 kV with a Hitachi HT7700 TEM (Hitachi, Tokyo, Japan). As for the control group, the same concentration of methanol was spotted on sterilized filter paper and the corresponding procedure was carried out as described for purified antifungal agents.

### Medium optimization for the production of antifungal agents.

To obtain a higher antifungal agent yield, single-factor tests were used to optimize the fermentation medium. First of all, the effects of three kinds of basic culture on the yield of antifungal active substances produced by bacterial strain *Bacillus* sp. wsm-1 were determined; these contained Luria-Bertani (LB) medium (tryptone at 10 g/liter, NaCl at 10 g/liter, yeast extract at 5 g/liter, distilled water at 1,000 ml [pH adjusted to 7.0]), nutrient broth (NB) medium (peptone at 10 g/liter, beef powder at 3 g/liter, NaCl at 5 g/liter, distilled water at 1,000 ml [pH adjusted to 7.0 to 7.4]), and Landy medium (glucose at 10 g, l-glutamic acid at 5 g, MgSO_4_·7H_2_O at 0.5 g, KCl at 0.5 g, KH_2_PO_4_ at 1.0 g, FeSO_4_·7H_2_O at 0.15 mg, MnSO_4_ at 5 mg, CuSO_4_·5H_2_O at 0.16 mg, distilled water at 1,000 ml). Fermentation and purification were carried out according to the method as described in “Isolation and purification of antifungal agents from *Bacillus* sp. wsm-1” above. Then, on the basis of the best base medium selected, the effects of different carbon sources (glucose, sorbitol, maltose, starch, sucrose, and fructose), nitrogen sources (peptone, sodium glutamate, yeast extract, soy peptone, ammonium sulfate, and tryptone) and amino acids (Pro, Tyr, Ser, Asn, Gln, and Cys) on the yield of antifungal agents produced by *Bacillus* sp. wsm-1 were detected. The fractions from HPLC were collected and air dried before measuring the weight, and then the yield of iturin W was calculated based on the weight of fractions from HPLC. All the experiments were carried out in triplicate, and the average yield of antifungal agents obtained was taken as the dependent variable.

## Supplementary Material

Supplemental file 1
