# Supplementary material for: Structural and Functional Insights into Iturin W, a Novel Lipopeptide Produced by the Deep-Sea Bacterium Bacillus sp. Strain wsm-1
Source: Appl Environ Microbiol. 2020 Oct 15;86(21):e01597-20. doi: 10.1128/AEM.01597-20 (PMC7580537; doi:10.1128/AEM.01597-20)
Supplement: Supplemental file 1 [file AEM.01597-20-s0001.pdf]

Supplementary information

Structural and functional insights of iturin W, a novel lipopeptide produced by the deep-sea bacterium *Bacillus* sp. wsm-1

Shengnan Zhou[a], Ge Liu[b,c,d] , Rikuan Zheng[b,c,d], Chaomin Sun[b,c,d*] , Shimei Wu[a*]

[a]College of Life Sciences, Qingdao University, Qingdao, 266071, China.

[b]CAS Key Laboratory of Experimental Marine Biology, Institute of Oceanology, Chinese Academy of Sciences, Qingdao, 266071, China

[c]Laboratory for Marine Biology and Biotechnology, Qingdao National Laboratory for Marine Science and Technology, Qingdao, 266071, China

[d]Center of Ocean Mega-Science, Chinese Academy of Sciences, Qingdao, 266071, China

[*] Corresponding author

Shimei Wu E-mail address: shimeiwu2016@126.com

Chaomin Sun E-mail address: sunchaomin@qdio.ac.cn

Keywords: *Bacillus*, biocontrol, antifungal, lipopeptide, iturin

Running title: A novel lipopeptide iturin W

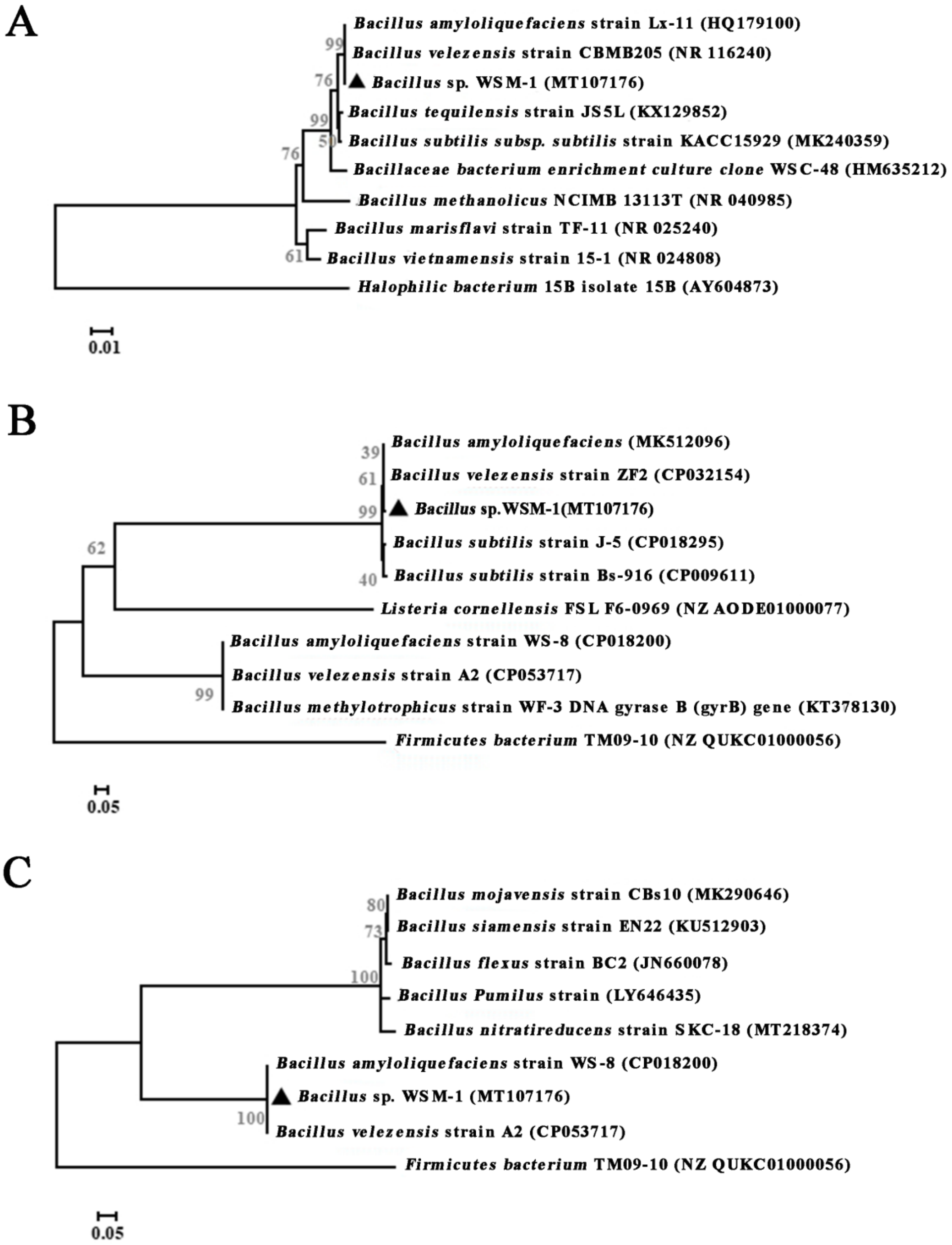

FIG S1 Neighbour-joining phylogenetic tree of *Bacillus* sp. wsm-1 and related species based on the sequence of 16S rRNA (A), *gyrB* (B) and *rpoD* (C) respectively. GenBank accession numbers are shown in parentheses. Bootstrap values (expressed as percentages of 1,000 replications) of >50 % are shown at branching nodes.
